# Gastrointestinal adenocarcinoma metastasizing to the vulva: a case report

**DOI:** 10.1186/s13256-017-1399-8

**Published:** 2017-08-22

**Authors:** Muchabayiwa F. Gidiri, M. Nyakura, M. Hukuimwe, T. Magwali, R. Makunike-Mutasa, F. D. Museta

**Affiliations:** 1 Academic Department of Obstetrics and Gynaecology, University of Zimbabwe College of Health Sciences, B-Floor, Old Health Sciences Building, Mazowe Street, Avondale, Harare Zimbabwe; 2 Department of Histopathology, University of Zimbabwe College of Health Sciences, Mazowe Street, Avondale, Harare Zimbabwe; 3 Department of Obstetrics and Gynaecology, Gaborone Private Hospital, P/BAG BR 130, Gaborone, Botswana

**Keywords:** Secondary vulval adenocarcinoma, Gastrointestinal origin, Metastatic, Vulva, Adenocarcinoma, Gastrointestinal primary

## Abstract

**Background:**

Metastatic vulval adenocarcinoma is a rare occurrence with only a few cases reported to date. They can arise from the breast, gastrointestinal system, or endometrium.

**Case presentation:**

We present the case of a 55-year-old Black African woman who presented with vulval itching which progressed to warty lesions. Histology revealed a vulval adenocarcinoma which immunohistochemistry suggested was of gastrointestinal origin. Colonoscopy later confirmed an anorectal tumor as the primary site. Despite extensive chest metastases she looked surprisingly well and had no pulmonary symptoms. The major source of symptomatic distress was the itchy extensive warty lesions on her vulva. She has since had a vulvectomy which gave her significant symptomatic relief.

**Conclusions:**

This case was interesting as vulval adenocarcinoma is a rare histological diagnosis found in less than 10% of vulval cancers. Primary vulval adenocarcinoma is rare with most of these cancers being secondary metastases from a distant site. Her symptoms were predominantly vulval with no chest symptoms even though she had extensive pulmonary metastases. She has been clinically well except for the itching suggesting an indolent course.

## Background

Cancer of the vulva has an incidence of approximately 3.3/100,000 in developed countries and is mostly seen in older women with a peak between 60 and 79 years of age. In older women the etiology is related to maturation disorders such as lichen sclerosis whereas in younger women it is seen in association with vulvar intraepithelial neoplasia (VIN) with evidence of oncogenic human papillomavirus (HPV). The incidence is predicted to increase as a result of an ageing population with more women living into old age [1]. The majority (approximately 50%) of these cancers are HPV related, although the correlation is not as strong as in VIN where HPV deoxyribonucleic acid (DNA) was only found in 13% of lesions [2, 3]. In Zimbabwe, according to the Zimbabwe National Cancer Registry, vulval cancer constitutes approximately 1.1% of all female cancers and 3.6% of all gynecological cancers [4]. The major cancer burden is with cervical cancer and by correlation we would suppose that there are more cases of vulval cancer in Zimbabwe than in developed countries as cervical cancer and vulval cancer are HPV related.

The histology is predominantly squamous cell in 90% of cases while the remaining 10% are a mixture of several other histological subtypes among them primary vulval adenocarcinoma usually of Bartholin glands. The histogenesis of primary vulval adenocarcinoma has not been fully understood; it mainly includes extramammary Paget’s disease, sweat gland carcinomas, and breast-like adenocarcinomas of the vulva [5]. Primary adenocarcinoma of the vulvar is extremely rare with approximately 13 cases reported to date, such that when a diagnosis is made there is always a suspicion of metastasis from a distant site such as the gastrointestinal tract [6]. Metastatic adenocarcinoma of the vulva is very rare with only a few cases reported to date.

## Case presentation

**Fig. 1:**
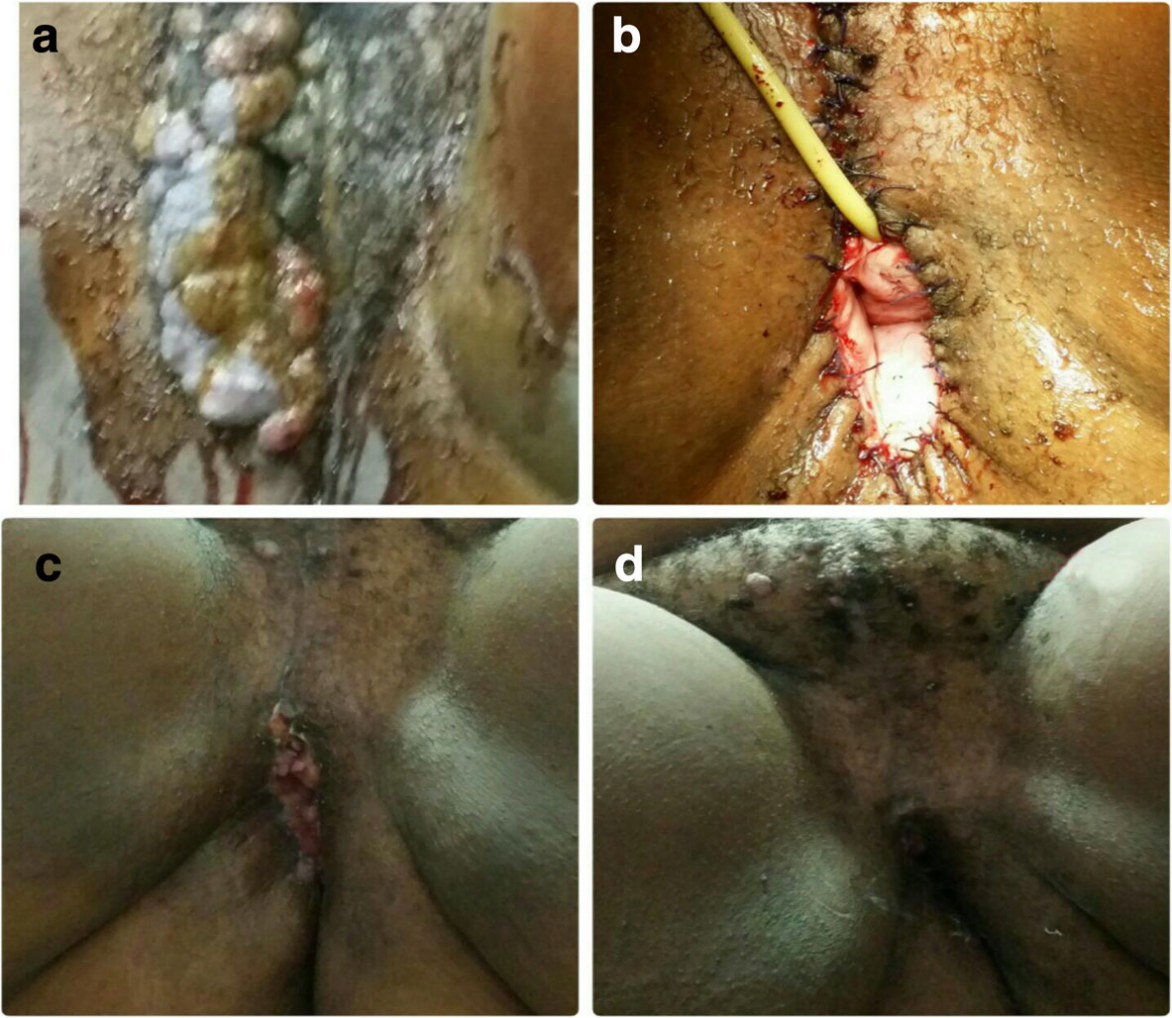
The vulva: **a** pre-operative; **b** immediately postoperative; **c** 1-month post operation and **d** 2-months post vulvectomy

We describe the case of a 55-year-old, para 7, postmenopausal black African woman who was initially seen with a history of vulval itching of 3 years’ duration and at that stage there were no lesions to report and the itchiness was treated with topical steroids with an initial good response. Seven months prior to seeing a gynecologist she noted three warty-like lumps on her right labia majora. She then presented to a gynecologist and an excisional biopsy was done. Histology surprisingly showed infiltrating moderate to well-differentiated adenocarcinoma with malignant glandular structures that were lined by tall columnar epithelium with stratification and mucin production. The warty lesions eventually became florid as shown in Fig. 1.a.

Since this was an unusual histological type for vulval cancer, immunochemistry was requested which showed mucin, strongly positive cytokeratin (CK) 20 and CDX2, and negative CK7. There was minimal patchy staining for p16 and features suggestive of large bowel or pancreatic primary tumor. Several years back, she had a hysterectomy for abnormal uterine bleeding and histology showed several leiomyomata and no evidence of uterine or cervical cancer. In view of the unusual histology, a mammography was done which showed a solitary benign lesion in her right breast. Computed tomography with contrast of her chest and abdomen showed multiple parenchymal shadows suggestive of metastasis in her lungs and there was rectal thickening of uncertain significance with no obvious large bowel or pancreatic lesions.

She underwent sigmoidoscopy; the findings were essentially normal. A biopsy of an enlarged left groin lymph node showed the presence of metastatic adenocarcinoma with an immunohistochemistry profile again consistent with spread from colonic or rectal adenocarcinoma. When she moved to Zimbabwe she presented to the gynecological clinic with a history of lumps on her vulva. These warty lesions were biopsied and sent for histology and immunohistochemistry. The results showed adenocarcinoma and immunohistochemistry again was suggestive of gastrointestinal malignancy as a primary. She was then commenced on chemotherapy; she received six cycles of oxaliplatin and capecitabine daily for 2 weeks with a 1-week break. After 4 months, the lesions, however, did not resolve, instead she developed a flare of the vulvar tumor associated with severe itchiness and pain.

A multidisciplinary team meeting involving gynecologists, general surgeons, radiologists pathologists, and radio-oncologists agreed to proceed with a colonoscopy and vulvectomy to alleviate her debilitating symptoms. A colonoscopy revealed anorectal cancer, and histology confirmed invasive adenocarcinoma of villoglandular type in keeping with anal rather than vulval origin. A vulvectomy was done and she commenced on radiotherapy. On clinical examination she was doing well and was relieved of her symptoms. Despite the chest metastases, she has never had significant pulmonary symptoms and she has always been clinically well except for the vulval symptoms.

Figure 1a shows vulva preoperatively, Fig. 1b shows the vulva immediately post operation, Fig. 1c shows the vulva 1 month post operation and Fig. 1d shows the vulva 2 months post vulvectomy.

## Discussion

Vulval adenocarcinoma can be primary arising *de novo* on the vulva but it is rare or it can be secondary following metastasis from a distant site. Vulval adenocarcinoma should be considered metastatic until proven otherwise [7, 8]. The various cancers that have been known to metastasize to the vulva are cervix, ovary, rectal, colon, endometrial, skin (malignant melanoma), breast, lung, vagina, anal, and bladder. Of all parts of the female genital tract, the vulva is the least frequent site of metastases [9]. Primary adenocarcinoma of the vulva is rare and is usually associated with origin from the Bartholin glands and primary Paget’s disease of the vulva. Most adenocarcinomas of the lower genital tract (cervix, vagina, and vulva) are related to HPV with the cervix being the commonest site [10]. The commonest histological subtypes of these are endocervical, intestinal, signet ring, and endometrioid. A large European study analyzing HPV prevalence in histology specimens showed that the commonest non-HPV-related cervical adenocarcinoma belonged to a spectrum of the gastric type.

There is a significant amount of literature that has been written about primary cervical but not vulval adenocarcinoma [8]. Most of these cervical intestinal and signet ring cell adenocarcinoma have been studied with immunohistochemistry and shown to be diffusely positive with CK7 and focally or diffusely positive with CK20 and CDX2 while being negative for p16 antigens. This is the evidence that they are probably not HPV related. These intestinal-type cervical adenocarcinomas are distinguished from metastases or from direct spread from a colorectal primary by a combination of clinical and pathological factors such as history, radiology, and pattern of cervical involvement. These are the same principles we applied to our case of vulval adenocarcinoma.

As alluded to earlier, primary vulval adenocarcinomas are rare most arising from primary vulval Paget’s disease or Bartholin glands. The World Health Organization (WHO) has classified these into Bartholin gland adenocarcinoma, adenocarcinoma of mammary gland type, adenocarcinoma of sweat gland origin, adenocarcinoma of Skene’s gland origin, and adenocarcinoma of intestinal type [11]. As in vaginal cancer, a metastasis should always be considered before making a diagnosis of primary vulval adenocarcinoma.

Metastatic vulval tumors are rare with only a few cases reported in the English literature. The largest series of metastatic tumors of the vulva was a clinicopathological study of 66 cases by Neto *et al*. [12]. They reviewed files of cases with metastatic vulval cancer from the Department of Pathology University of Texas. Some histology specimens were also reviewed. They found that in 3 out of 66 (4.5%) cases the primary tumor was from the colon of which one was synchronous with the primary and the other two developed at intervals of 24 and 84 months after the primary cancer had been diagnosed. The other sites of primary gastrointestinal tumor were rectal (seven cases) and pancreatic (one case). The biggest contributor to metastases in this series was the cervix (22%) contributing 15 cases followed by ovarian cancer (8 cases). In three cases vulval metastases were diagnosed before the primary. Besides vulval metastases, 93% of patients in this series also had simultaneous metastases to other sites. A total of 21 out of 62 (34%) cases had pulmonary metastases as well. Rectal adenocarcinoma was the third commonest primary after cervical and ovarian cancer. The commonest site of metastasis on the vulva was the labia majora in 66% of cases. Various treatment modalities in different combinations including wide local excision, chemotherapy, radiotherapy, and vulvectomy were associated with varying survival benefits, on average 7.5 months from diagnosis.

We managed to identify the primary as anorectal in our case with metastases to the vulva and lungs although we could not biopsy the pulmonary lesions. It is important to know that primary vulval adenocarcinomas, although rare, do exist and must be differentiated from secondary tumors through histology and immunohistochemistry. Palliative surgery and radiotherapy were perhaps the best modalities for our patient.

## Conclusions

Vulval adenocarcinoma is a rare histological diagnosis and on diagnosis an effort should be made to identify the primary tumor. Primary vulval adenocarcinoma can arise from the Bartholin’s gland but is rare. Immunohistochemistry is useful in differentiating several types of adenocarcinoma. Treatment is often palliative with a few months’ survival from diagnosis.
